# Multi-Scale Gradient Fiber Structure Hierarchical Flexible Ceramic Aerogel for High-Temperature Filtration

**DOI:** 10.3390/nano16060382

**Published:** 2026-03-23

**Authors:** Chuan-Hui Guo, Yuan Gao, Chao Zhang, Chu-Bing Li, Yue-Han Sun, Hong-Xiang Chu, Run-Ze Shao, Zhi-Wei Zhang, Yun-Ze Long, Jun Zhang

**Affiliations:** Collaborative Innovation Center for Nanomaterials & Devices, College of Physics, Qingdao University, Qingdao 266071, China; guochuanhui@qdu.edu.cn (C.-H.G.); 2021010006@qdu.edu.cn (Y.G.); 2025010124@qdu.edu.cn (C.Z.); lichubing@qdu.edu.cn (C.-B.L.); sunyuehan@qdu.edu.cn (Y.-H.S.); chuhongxiang@qdu.edu.cn (H.-X.C.); shaorunze@qdu.edu.cn (R.-Z.S.); zhangzhiwei@qdu.edu.cn (Z.-W.Z.)

**Keywords:** air-blown spinning, ceramic nanofiber aerogels, air filtration, high-temperature resistance

## Abstract

High-temperature particulate matter (PM) filtration remains a fundamental challenge, because most fiber filters not only face the challenge of high temperatures but also suffer from an inherent trade-off between capture efficiency, pressure drop, and service life. This paper reports a hierarchical layered zirconia (ZrO_2_) ceramic fiber aerogel featuring a continuous multiscale gradient. The aerogel was prepared by gradient air-blown spinning, and the resulting structure has directional order, with the fiber diameter gradually decreasing from upstream to downstream, thus forming a pore size gradient and achieving hierarchical particle interception across multiple scales. This rational design simultaneously suppresses surface clogging and reduces flow resistance, resolving the longstanding trade-off between efficiency and permeability. Consequently, this aerogel achieves an ultra-high filtration efficiency of 99.96%, a low pressure drop of 156 Pa, and a high dust-holding capacity of 101 g m^−2^. The material also exhibits outstanding mechanical toughness (80% compressive strain elasticity and 25.75% tensile fracture strain) and thermal stability up to 1000 °C. Moreover, it maintains over 99.95% filtration efficiency at high temperatures and can be fully regenerated through 800 °C heat treatment. This work establishes a structure-based design paradigm for high-temperature filtration media and provides a scalable pathway for next-generation industrial flue gas purification.

## 1. Introduction

The rapid development of cities and industries has led to significant particulate matter (PM) pollution in the air, severely impacting the global environment and posing substantial threats to human health [1,2]. Inhalable fine particles can trigger cardiovascular diseases, respiratory disorders, and may even contribute to lung cancer [3,4,5,6]. PM mainly originates from high-temperature emissions, such as coal combustion, industrial activities, and vehicle exhaust [7]. In recent years, stricter environmental and health regulations have made the direct removal of impurities from high-temperature gas sources essential for environmental protection and human health [8]. This has generated increasing interest in high-temperature gas filtration. Traditional inorganic filters are unsuitable for high-temperature applications due to their heavy weight, poor filtration efficiency, and inferior mechanical properties. Fiber filters exhibit higher porosity, and nanofibers demonstrate a “sliding effect” that reduces airflow resistance. As a result, fiber filters achieve higher filtration efficiency and lower pressure drops [9,10]. Filtration efficiency improves as nanofiber diameter and filter pore size decrease [11,12]. Therefore, thermally stable nanofiber materials, such as ceramic fibers, melt-blown polyester, or polyimide fibers, are commonly used to remove PM from the source via mechanisms including direct interception, inertial impaction, electrostatic deposition, Brownian diffusion, and gravitational settling [13,14]. Ceramic fibers, due to their inert chemical composition, exhibit superior chemical stability and are often fabricated as porous membranes or fiber films for high-temperature source filtration [8,15,16]. Electrospinning is a widely used method for producing ceramic fibers. Electrospun ceramic nanofiber membranes effectively collect PM due to their small fiber diameters, tunable porous structures, and ease of fabrication from various materials [17,18,19,20]. However, electrospun air filtration materials often exhibit high pressure drops due to the dense packing of nanofibers (limited porosity, <90%), resulting in significant energy consumption [21]. Furthermore, due to their inherent anisotropic layered deposition characteristics, electrospun nanofiber membranes are limited to a thickness of typically <100 μm, resulting in poor dust-holding capacity during prolonged filtration [22]. Therefore, there is a need to develop more efficient, energy-saving, and durable filtration materials.

Nanofiber aerogels (NFAs) offer several structural advantages, including a large specific surface area, high porosity, abundant interconnected channels, and the ability to achieve substantial thickness, making them more promising for high-temperature air filtration than nano-ceramic fiber membranes [23,24,25]. The manufacturing techniques for aerogels used in filtration applications mainly include freeze-drying and air-blown spinning technologies. Recently, a honeycomb-like elastic fiber aerogel with a gradient structure was fabricated using directional freeze-drying. This aerogel exhibits excellent mechanical properties and filtration performance [26,27]. However, directional freeze-drying is relatively complex, involving challenges in structural control and shaping, as well as long processing times, which limit its suitability for large-scale production. In contrast, air-jet spinning offers advantages such as a simple process, high fiber production efficiency, and broad material compatibility, and the resulting fiber aerogels also exhibit excellent filtration performance [28,29]. However, nanofiber aerogels produced by air-blown spinning typically exhibit relatively uniform fiber sizes and simple structural features, which limit further improvements in filtration performance. Consequently, achieving simultaneous optimization of mechanical robustness and filtration efficiency through structural design of nanofiber aerogels via air-blown spinning remains a significant challenge.

In this study, we prepared a ZrO_2_ ceramic fiber aerogel with a layered structure and multiscale fiber gradient distribution (gZCFA) using a flow-gradient-driven air-blown spinning method. gZCFA shows a continuous gradient in fiber diameter from thick to thin in the vertical direction, with fibers arranged in a layered structure in the parallel direction. gZCFA demonstrates superior filtration efficiency (99.96%) and a low pressure drop (156 Pa), maintaining excellent performance even for high-temperature gases. Additionally, gZCFA can withstand high tensile and compressive strains without undergoing plastic deformation. Its unique fiber structure provides high filtration performance, extended service life, and superior mechanical properties, making it highly suitable for high-temperature flue gas filtration.

## 2. Materials and Methods

### 2.1. Materials

Zirconium acetate with 23% purity was bought from Shanghai Adamas Reagents Co., Ltd., Shanghai, China. PVA-1788 and AlCl_3_·6H_2_O (99%) were obtained from Shanghai Aladdin Reagent Co. Ltd., China. Phosphoric acid (H_3_PO_4_, AR) and tetraethyl orthosilicate (TEOS, AR) were supplied by China National Shanghai Pharmaceutical Group Chemical Reagent Co., Ltd. Pure water was obtained from Shanghai McLean Biochemical Co., Ltd, China.

### 2.2. Preparation of the Spinning Solution for ZrO_2_

The spinning solution was prepared as follows: First, PVA-1788 was added to pure water to prepare a 15 wt% PVA aqueous solution. Subsequently, 4.387 g of TEOS and 0.03 g of H_3_PO_4_ were added to 22.44 g of the PVA aqueous solution, and the mixture was stirred thoroughly at room temperature. A quantity of 2.54 g of AlCl_3_·6H_2_O was added to the specified solution, and the mixture was stirred at room temperature for 60 min until complete dissolution was achieved. Finally, 22.5 g of zirconium acetate was added to the clear solution. Following a stirring process at ambient temperature for 1 h, a sonication step was performed for 30 min to yield a transparent, clear sol–gel solution that served as the spinning solution.

### 2.3. Preparation of gZCFA

A 10 mL syringe equipped with a needle (30 G gauge, 6.5 mm long, inner diameter 0.21 mm, outer diameter 0.41 mm) was filled with the spinning solution. An injection pump was used to deliver the solution to the syringe needle at a rate of 8 mL·h^−1^. By adjusting the air compressor pressure, different fiber diameters were obtained by sweeping the solution with varying airflow velocities. The spinning air pressure ranged from approximately 0.25 bar to 1.25 bar, with each pressure adjustment increment set at about 0.1 bar. The distance between the nozzle and the porous cage collection device was approximately 60 cm. The 24 cm diameter cage had a sealed bottom and 1 cm square holes on the sides. Furthermore, the environmental temperature during the airflow spinning process was regulated at 25 ± 5 °C, with ambient humidity maintained at 35 ± 5%. The resultant precursor of the gradient ceramic nanofiber aerogel was then placed in a muffle furnace. The temperature was raised at 2 °C·min^−1^ to 1100 °C, held at 1100 °C for 60 min to remove organic components, then cooled in the furnace to obtain gZCFA.

### 2.4. Preparation of cZCFA and fZCFA

The preparation method is consistent with that of gZCFA, with only the air compressor pressure adjusted to control the gas flow velocity. The gas flow pressure for preparing cZCFA is approximately 0.75 bar, while that for fZCFA is approximately 1.25 bar.

### 2.5. Characterization

At room temperature, the viscosity of the solution was measured using a rotational viscometer (LV-SSR, FangRui, Shanghai, China). A quantity of the spinning solution was poured into the sample cup, and three measurements were made to obtain the average viscosity value. Under different air pressures, the airflow velocity was measured at a distance of 2 cm using a wind pressure and air volume meter (SG-312, Reanow, Shanghai, China). Three measurements were taken, and the average value was recorded. A total of 50 randomly selected fibers were measured for their diameters. This was performed using the image analysis software Nano Measurer 1.2, and the diameter distribution thus obtained was subsequently subjected to statistical evaluation. gZCFA density and porosity were measured as per a known method [30]. The microstructure and elemental distribution of gZCFA were observed using a field emission scanning electron microscope (FE-SEM, Sigma 500, Zeiss, Aubergen City, Baden-Württemberg State, Germany) equipped with energy-dispersive X-ray spectroscopy (EDS). XRD patterns for the samples were recorded using an X-ray diffractometer (Smartlab, Rigaku, Tokyo, Japan) equipped with Cu Kα radiation and a 2θ range of 10–80°, with a scanning rate of 5° min^−1^. An X-ray photoelectron spectrometer (XPS, Escalab Xi+, Thermo Fisher, Waltham, MA, USA) equipped with a 300 W Al Kα radiation source was utilized for the analysis of the elemental composition of gZCFA. An investigation was undertaken to examine the mechanical properties of gZCFA, utilizing a universal testing machine (Instron 5300, Boston, MA, USA) equipped with a 100 N load cell. Tensile and compression tests employed strain rates of 2 mm min^−1^ and 5 mm min^−1^, respectively. Cyclic compression tests utilized a strain rate of 200 mm min^−1^. Thermal stability was measured in air using a thermogravimetric analyzer (TGA, Netzsch STA 449 F3, Bavaria, Germany) over the temperature range of 30 to 1000 °C at a heating rate of 10 °C min^−1^.

### 2.6. Filter Test

Room-temperature filtration: The filtration performance of gZCFA was tested using a filter tester (MFP 3000, PALAS, Baden-Württemberg State, Germany). Particles derived from a 10 wt% KCl solution in water were used as the particle source, employing a filtration area of 100 cm^2^. The quality factor (*QF*) was calculated using the following formula:

(1) $$QF=\frac{\left[-\ln\left(1-\eta \right)\right]}{△P}$$

Here, *η* and Δ*P* represent filtration efficiency and pressure drop, respectively.

High-Temperature Filtration: The experimental procedure comprised the independent conceptualization and construction of a filtration test apparatus operating at elevated temperatures. Quartz tubes served as the filtration tubes. A tube furnace provided the high-temperature conditions, with particulate matter generated by incense burning. The airflow was provided by the air compressor, and the airflow rate was regulated by the flowmeter. A particle detector (DT-9880 M, CEM, Shenzhen, China) measured the particle concentration at the inlet and outlet in accumulation mode, with a sampling duration of 30 s. In addition, the pressure drop was measured and recorded using a digital differential pressure meter (HT-1891). Filtration efficiency was then determined using the following formula:

(2) $$\eta =N_{1}-\frac{N_{2}}{N_{1}}$$

Here, *η* is the filtration efficiency. *N*_1_ and *N*_2_ are the particle counts at the air inlet and outlet, respectively.

## 3. Results and Discussion

### 3.1. Manufacturing and Filtration Principles of gZCFA

**Figure 1:**
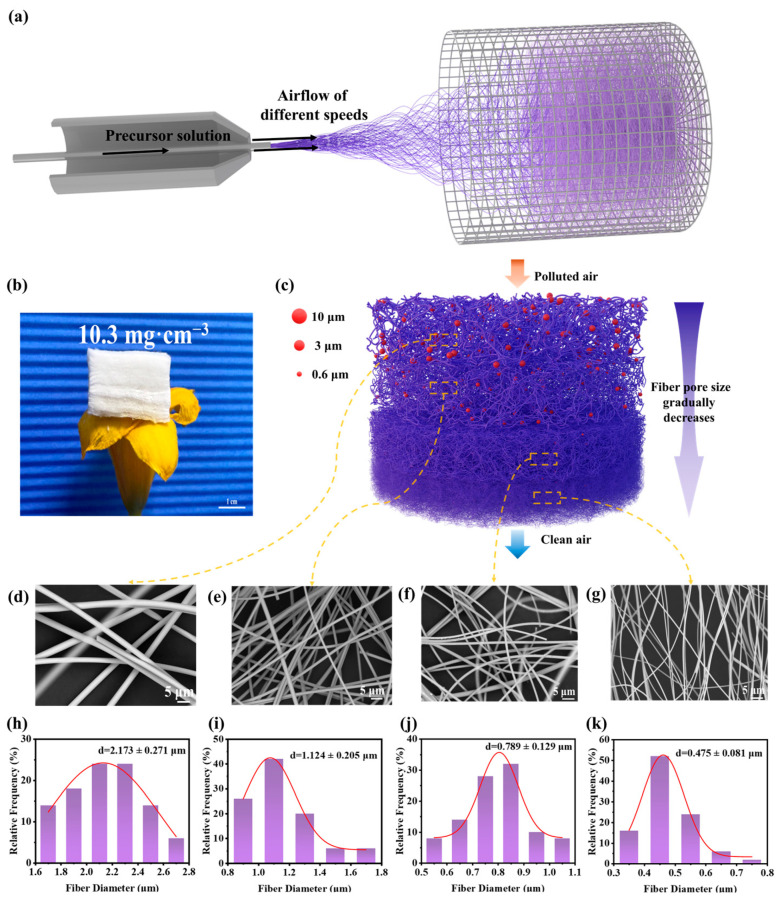
Manufacturing and filtration principles of gZCFA. (**a**) Manufacturing flowchart for gZCFA. (**b**) Optical image of gZCFA on a yellow petal. (**c**) Illustration of gZCFA’s particle removal mechanism as an air filtration material. (**d**–**g**) SEM images of the four fibers in gZCFA. (**h**–**k**) Fiber diameter distribution curves for the four fibers.

We used the flow-gradient air-blown spinning method to efficiently prepare gZCFA. As shown in Figure 1a, the preparation process consists of an automatic syringe pump, an air pump, and a cage-shaped collection device. The syringe pump continuously extruded the precursor solution (viscosity: 690 cP) through the needle. The air pump pressure was continuously adjusted to generate high-speed airflow, which swept the spinning solution at varying velocities. As the solution converged at the needle tip, shear forces from the airflow caused the jet to exhibit unstable motion within a turbulent field. Solvent evaporation occurred, leading to the solidification of the solute, which was then deposited onto the collection device, forming zirconia precursor fibers with a three-dimensional structure (Figure S1). The precursor fibers were subjected to calcination at 1100 °C for 1 h, a process that resulted in the removal of the polymer template. This process was undertaken to yield gZCFA, which possessed a low density (approximately 10.3 mg·cm^−3^) and a high porosity (99.1%). As shown in Figure 1b, gZCFA stands upright on yellow petals without deformation, and cross-sections reveal a layered structure. Figure S2 further confirms the layered characteristics of gZCFA. The microarchitecture of gZCFA was characterized by field emission scanning electron microscopy (FE-SEM), which revealed a layered fiber arrangement (Figure S3). Notwithstanding the elevated calcination temperature, the fibers exhibited a smooth surface morphology, thereby suggesting the absence of grain growth (Figure S4a). The composition and distribution of elements within the gZCFA fibers were revealed by EDS (Figures S4b and S5). gZCFA primarily consists of zirconium (Zr), aluminum (Al), silicon (Si), and oxygen (O), with a uniform elemental distribution. In order to characterize the composition and crystallinity of gZCFA, X-ray photoelectron spectroscopy (XPS) and X-ray diffraction (XRD) were employed as analytical methods (Figure S6a). As demonstrated in Figure S6b, analysis of the binding energies of ZrO_2_ revealed the presence of Zr 3d_3_/_2_ and Zr 3d_5_/_2_ components, with respective energy levels of 184.5 eV and 182.1 eV. These findings confirm the presence of the +4 oxidation state of Zr and substantiate the observation of the tetragonal phase of ZrO_2_ [31,32,33]. Si 2p (102.9 eV) and Al 2p (74.4 eV) peaks indicate the presence of aluminosilicate. The peaks at 532.2 eV and 530.2 eV indicate the presence of lattice oxygen. The 119 eV Al 2s peak confirms the presence of α-Al_2_O_3_ [34]. Figure S7 demonstrates that XRD analysis revealed that gZCFA exhibits a highly crystalline tetragonal zirconia (t-ZrO_2_) structure, consistent with XPS analysis.

The structural design of fiber filter materials plays a key role in their filtration performance. We used the flow-gradient air-blown spinning method with a gradual flow velocity gradient to synthesize gZCFA. During filtration, the layered structure arranges most fibers perpendicular to particle movement, improving particle interception efficiency [35]. As fiber diameter decreases, pore tortuosity increases, reducing the filter’s mass flow rate and facilitating easier particle capture [36,37]. Therefore, fiber aerogels with a layered gradient structure are more likely to achieve staged filtration while balancing filtration efficiency and pressure drop. Figure 1c illustrates the filtration mechanism of gZCFA. As contaminated air flows through the gZCFA, coarse fibers in the upper layer intercept large particles, primarily PM_10_, while smaller particles are carried by the airflow to the next layer, preventing excessive particle accumulation on the surface. Similarly, thicker fibers in the middle layer capture PM_3_, while the thinnest fibers in the bottom layer intercept smaller particles (PM_0.6_), enabling multi-stage filtration. The upper fibers of gZCFA capture larger particles, reducing surface particle density and mitigating the skin effect. This also reduces the load on the lower fibers, which intercept smaller particles. Consequently, the material achieves high filtration efficiency with a low pressure drop. SEM observations and fiber diameter measurements were conducted on the gZCFA fiber assembly, with four representative fibers selected (Figure 1d–g). gZCFA consists of ZrO_2_ fibers of varying diameters, showing a gradual diameter change from top to bottom. The top-layer fibers measure 2.173 μm, while the bottom-layer fibers measure 0.475 μm. Intermediate layers have fiber diameters of 1.124 μm and 0.789 μm, respectively (Figure 1h–k). In addition, the density and porosity of materials containing four different fiber sizes were measured and calculated, respectively. As shown in Figure S8, the gradual reduction in fiber diameter leads to an increase in material density, accompanied by a corresponding decrease in porosity and pore size. Therefore, the density and porosity of gZCFA are nonuniform, indicating a gradual variation along the gradient direction. According to filtration theory and the single-fiber efficiency (SFEE) model [36,38,39],

(3) $$Et=1-\exp\left[\frac{4V_{f}El_{0}}{\pi \left(1-V_{f}\right)d_{f}}\right]$$

where *E* is the efficiency of a single fiber, *V_f_* is the fiber volume fraction, *d_f_* is the fiber diameter, and *l*_0_ is the filter thickness. As fiber diameters decrease, their filtration efficiency increases, and the dominant filtration mechanism shifts from physical interception to diffusion. gZCFA contains fibers of multiple scales, enabling it to employ multiple filtration mechanisms. Consequently, gZCFA demonstrates superior filtration performance compared to materials composed of a single fiber scale. Furthermore, the gradient in gZCFA fiber diameters induces a corresponding gradient in pore sizes, enabling simultaneous optimization of filtration efficiency and pressure drop.

### 3.2. Mechanical Properties of gZCFA

**Figure 2:**
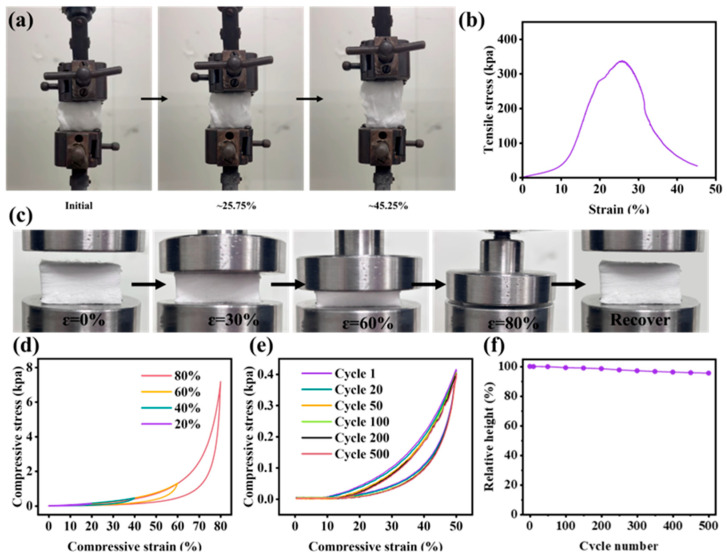
Mechanical properties of gZCFA. (**a**) Optical image of gZCFA under tensile stress at room temperature. (**b**) Stress–strain curve of gZCFA under tensile stress at room temperature. (**c**) Optical image of gZCFA subjected to uniaxial compression up to 80% strain. (**d**) Stress–strain curves of gZCFA under uniaxial compression at various compression ratios. (**e**) Stress–strain curve of gZCFA after 500 compression cycles at 50% strain. (**f**) Relative height of gZCFA after 500 compression cycles.

gZCFA exhibits exceptional thermal resistance due to its ZrO_2_ ceramic fiber composition. As shown in Figure S9, thermogravimetric analysis indicates that the mass of gZCFA remains nearly constant as the temperature increases from room temperature to 1000 °C. Ceramic fibers provide gZCFA with high heat resistance and excellent mechanical properties. Moreover, the layered structure of gZCFA further enhances its mechanical properties. An evaluation of the mechanical properties of gZCFA was conducted utilizing a universal testing machine. Tensile stress–strain tests were performed on gZCFA at room temperature (Figure 2a,b). The results show a fracture strain of approximately 25.75% and a maximum tensile stress of about 335 kPa, demonstrating excellent ductility. As the strain increased to 45.45%, gZCFA exhibited non-brittle fracture behavior. This is due to the interaction between nanocrystals and the glass phase within ZrO_2_ fibers, along with the effective entanglement between fibers, which efficiently distributes tensile stress during stretching [40]. The amorphous phase serves to mitigate surface and pore defects at grain boundaries, while its viscous creep behavior and the resultant effective suppression of crystallite sliding collectively endow the polycrystalline ceramic fibers with superior deformability and satisfactory strength, alongside an improvement in material flexibility [41]. Compression testing was then performed. As demonstrated in Figure 2c,d, gZCFA withstood 80% compressive strain and quickly returned to its original dimensions after the removal of external force. To assess the compressive fatigue performance of gZCFA, load–unload fatigue testing was conducted at a compression ratio of ε = 50% for a total of 500 cycles (Figure 2e). The superior compression stability of gZCFA under cyclic compressive loading can be attributed to the effective formation of a nanofibrous network, which substantially mitigates plastic deformation during the compression process. After 500 compression cycles, the height of gZCFA remained nearly unchanged, exhibiting only approximately 5% permanent deformation (Figure 2f). The excellent compression stability of gZCFA under cyclic loading is attributed to its well-constructed nanofibrous network, which effectively suppresses plastic deformation during compression.

### 3.3. Filtration Performance of gZCFA

The filtration performance of gZCFA was tested using a PALAS filtration instrument (Figure S10). To verify that gZCFA represents the optimal fiber combination, we conducted filtration performance tests on sample groups with varying fiber diameters: sample group 1 (2.17 μm), sample group 2 (2.17 μm and 1.12 μm), sample group 3 (2.17 μm, 1.12 μm, and 0.78 μm), sample group 4 (2.17 μm, 1.12 μm, 0.78 μm, 0.47 μm, and 0.38 μm), and gZCFA, all under the same surface density (22 mg·cm^−2^) and airflow rate (Figure S11). The results indicated that adding fibers of 0.38 μm diameter did not significantly improve filtration efficiency but caused a rapid increase in pressure drop. Therefore, from the perspective of filtration performance, gZCFA exhibits both high filtration efficiency and low pressure drop, resulting from its optimal fiber combination. To assess the impact of gZCFA’s layered gradient structure on filtration performance, we prepared a non-gradient coarse zirconia ceramic fiber aerogel (cZCFA) and a fine zirconia ceramic fiber aerogel (fZCFA) and compared their performance with gZCFA. Both cZCFA and fZCFA were prepared using solution jet spinning to form zirconia ceramic fiber aerogels with layered structures, but with fibers of a single scale. The average fiber diameter of cZCFA was 1.156 μm, while that of the thin fibers in fZCFA was 0.479 μm (Figure S11). For a comprehensive comparison, we evaluated the three materials at four different areal densities (10.7 mg·cm^−2^, 16.4 mg·cm^−2^, 22.5 mg·cm^−2^, 28.2 mg·cm^−2^), which were controlled by adjusting the spinning time.

**Figure 3:**
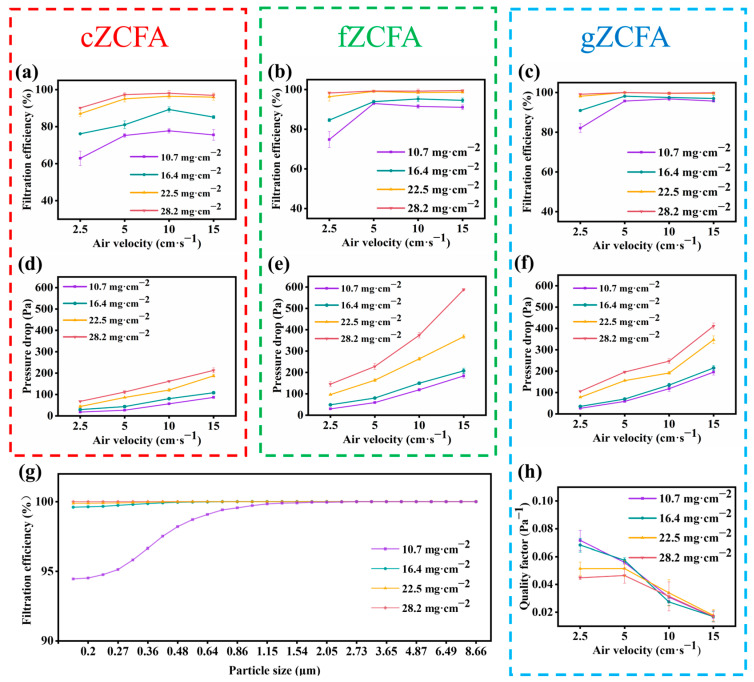
Filtration performance of gZCFA, cZCFA, and fZCFA. Filtration efficiency of the three materials at various areal densities and airflow velocities. The red dashed box and the green dashed box respectively represent the filtration performance of cZCFA and fZCFA, including filtration efficiency and pressure drop. The blue dashed box represents the filtering performance of gZCFA, including filtration efficiency, pressure drop and quality factor. (**a**) cZCFA. (**b**) fZCFA. (**c**) gZCFA. Pressure drop of the three materials at different areal densities and airflow velocities. (**d**) cZCFA. (**e**) fZCFA. (**f**) gZCFA. (**g**) Filtration efficiency of gZCFA at different areal densities for particles of various sizes at 5 cm·s^−1^. (**h**) Quality factor of gZCFA at different areal densities and airflow velocities.

The filtration efficiency of the three materials as a function of areal density and airflow velocity is shown in Figure 3a–c. The filtration efficiency of cZCFA, fZCFA, and gZCFA increases with areal density. At areal densities of 10.7 mg·cm^−2^ and 16.4 mg·cm^−2^, the relatively low material thickness resulted in lower filtration efficiencies for all three. However, at areal densities of 22.5 mg·cm^−2^ and 28.2 mg·cm^−2^, the filtration efficiencies of all three materials significantly improved. Specifically, for gZCFA, the filtration efficiency increased from 95.69% to 99.98% as areal density increased from 10.7 mg·cm^−2^ to 28.2 mg·cm^−2^ (Figure 3c). The effect of airflow velocity on filtration efficiency can be categorized based on areal density. At low areal densities, filtration efficiency for all three materials depends strongly on airflow velocity. At 10.7 mg·cm^−2^ and 16.4 mg·cm^−2^, filtration efficiency increases with airflow velocity. At high areal densities, airflow velocity had a minimal impact on filtration efficiency for all three materials. Notably, gZCFA at 28.2 mg·cm^−2^ maintained stable efficiency at high velocities, retaining 99.84% efficiency at 15 cm·s^−1^ (Figure 3c). The fiber assembly of gZCFA has a multiscale gradient structure that combines thick and thin fibers, enabling superior filtration efficiency. Additionally, gZCFA demonstrates excellent efficiency for fine particulate matter (Figure 3g).

Pressure drop is a key indicator of filter performance, with lower values preferred as higher airflow resistance increases energy consumption during filtration. As shown in Figure 3d–f, the pressure drop of all three materials increases with areal density. At an airflow velocity of 5 cm·s^−1^, the pressure drop of gZCFA increased from 59 Pa to 196 Pa as areal density increased from 10.7 mg·cm^−2^ to 28.2 mg·cm^−2^ (Figure 3f). Additionally, the pressure drop of all three materials depends on both areal density and airflow velocity. As airflow velocity increases, the pressure drop increases nearly linearly. When airflow velocity increased from 2.5 cm·s^−1^ to 15 cm·s^−1,^ the pressure drop of gZCFA with a surface density of 22.5 mg·cm^−2^ rose from 78 Pa to 347 Pa (Figure 3f). This proportional relationship between pressure drop and airflow velocity follows Darcy’s Law [42]. At all airflow velocities, the pressure drop of gZCFA across all four areal densities was lower than that of fZCFA under equivalent conditions. Although cZCFA exhibited a low pressure drop, its filtration efficiency was inferior. The fiber diameters in gZCFA span multiple scales, forming a unique layered gradient structure. This structure maintains a filtration efficiency comparable to that of fZCFA, while also sustaining a relatively low pressure drop (Figure 3f). To better understand the filtration performance of gZCFA, the quality factor (QF) for gZCFA with varying surface densities at different airflow rates was calculated (Figure 3h) and compared with those of cZCFA and fZCFA (Figure S12). gZCFA exhibits a favorable quality factor that decreases as airflow velocity increases (Figure 3h).

Thanks to its unique layered gradient structure, gZCFA enables staged filtration of particulate matter. The coordination of multi-scale fibers not only reduces the skin effect but also compensates for the limited filtration efficiency. The incorporation of coarse fibers forms more tortuous flow channels, thereby reducing airflow resistance during filtration, while the addition of fine fibers decreases pore size and enhances particulate capture efficiency. The gradient arrangement of fibers with varying diameters in gZCFA generates a corresponding pore-size gradient from large to small throughout the material, thereby contributing to its excellent air filtration efficiency. As shown in Figure S14, the filtration performance of gZCFA slightly decreased under low-humidity conditions but declined significantly under high humidity. This deterioration is likely caused by the formation of a thin water film on the fiber surfaces, which weakens electrostatic interactions and partially blocks the pores of the material. Consequently, with increasing humidity, the filtration efficiency decreases while the pressure drop increases. gZCFA not only achieves a high filtration rate but also maintains low airflow resistance, making it an efficient filter [43]. Furthermore, the filtration performance of gZCFA exceeds that of cZCFA and fZCFA, which feature a layered structure but lack a gradient architecture, highlighting the advantages of the layered gradient structure.

### 3.4. High-Temperature Filtration Performance of gZCFA

**Figure 4:**
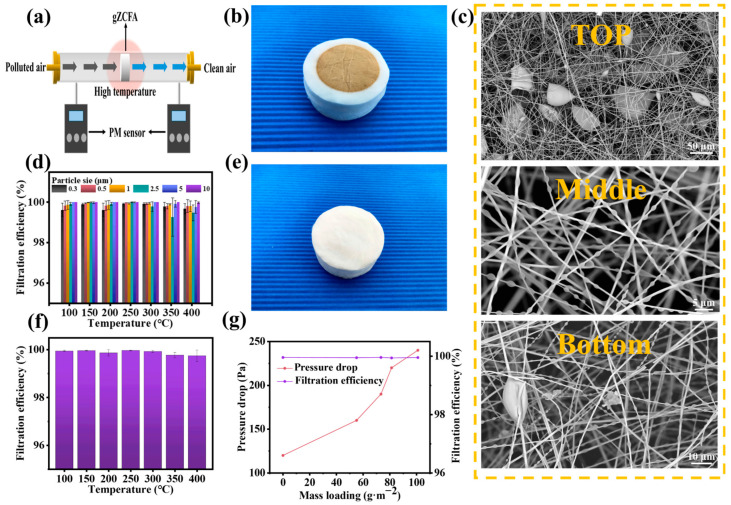
High-temperature filtration performance testing. (**a**) Schematic diagram of the high-temperature filtration device. The arrows in the figure indicate the direction of the airflow. (**b**) Optical image of gZCFA after high-temperature filtration. (**c**) SEM images showing particle distribution across the top, middle, and bottom layers of gZCFA. (**d**) Filtration efficiency of gZCFA for PM_0.3–10_ at varying temperatures. (**e**) Optical image of gZCFA after 1 h of heat treatment at 800 °C following high-temperature filtration. (**f**) Filtration efficiency of gZCFA at different temperatures. (**g**) Filtration efficiency and pressure drop of gZCFA during long-term filtration.

It is acknowledged that the waste gas emanating from industrial activities and transportation is characteristically high in temperature. The present study, therefore, examined the filtration performance of gZCFA under such conditions. We built an experimental apparatus to evaluate the high-temperature filtration capabilities of gZCFA (Figure 4a and Figure S13). Particulate matter generated from incense combustion was used as the pollution source, and its particle size distribution is shown in Figure S14. As shown in Figure 4b, the optical image of gZCFA after high-temperature filtration shows the capture of a large amount of particulate matter. Following heat treatment at 800 °C for 1 h, gZCFA returned to its original state (Figure 4e), demonstrating that the captured particulate matter can be removed through heat treatment, enabling reuse. Additionally, the fatigue resistance of gZCFA was evaluated (Figure S17). After 20 dust saturation cycles followed by heat treatment, the filtration efficiency of gZCFA decreased slightly. These results indicate that gZCFA possesses excellent thermal stability and reusability while maintaining high filtration efficiency. SEM was used to observe the top, middle, and bottom sections of gZCFA after high-temperature filtration. As shown in Figure 4c, the top-layer fibers primarily capture larger particles, the middle-layer fibers capture both larger and smaller particles, and the bottom-layer fibers mainly capture smaller particles, enabling multi-stage filtration. Since high-temperature exhaust gases typically do not exceed 400 °C and particles are prone to thermal decomposition at excessively high temperatures, we tested the filtration performance of gZCFA (areal density: 22 mg·cm^−2^) within this temperature range. Experimental results show that gZCFA effectively filters particulate matter at all temperatures (Figure 4d,f). The filtration efficiency for PM_0.3–10_ is at least 99.95%, and for PM_0.3_, it exceeds 99.92%.

Dust-holding capacity is a critical parameter for air filters, reflecting their service life. We tested the filtration efficiency and pressure drop variation in gZCFA (surface density: 22.5 mg·cm^−2^) at a flow velocity of 5 cm·s^−1^ (Figure 4g). The initial filtration efficiency of gZCFA was 99.954%, and the pressure drop was 120 Pa. After prolonged mass loading, the pressure drop doubled to 240 Pa, while the filtration efficiency remained at 99.951% (mass loading: 101 g·m^−2^). The service life of an air filter is typically defined as the time for its pressure drop to double from the initial value [13]. The pressure drop versus mass loading function in Figure 4g indicates that gZCFA has a long service life and high dust-holding capacity.

Finally, the filtration performance of gZCFA was compared with that of other flexible air filtration materials [24,26,27,29,44,45,46,47,48,49,50,51,52] (Table S1). The maximum operating temperature of both traditional polymer filters and polymer-based composite filtration materials does not exceed 400 °C. In contrast, gZCFA exhibits a significantly higher operating temperature, making it more suitable for high-temperature filtration applications. Compared with conventional ceramic fiber filter materials, gZCFA achieves high filtration efficiency while maintaining a low pressure drop, owing to its unique gradient layered structure, thereby exhibiting superior overall filtration performance. Compared with commercial filter materials (SiC), gZCFA has the characteristics of low weight and flexibility, which confer advantages in certain application scenarios, such as when there are requirements for filter weight or when there is compression deformation. Compared with other three-dimensional multi-scale gradient fiber aerogel filtration materials, gZCFA exhibits superior filtration performance and enhanced thermal resistance, making it particularly suitable for high-temperature filtration applications.

## 4. Conclusions

In summary, our air-blown spinning method, based on a flow velocity gradient, enables the efficient preparation of fiber aerogels with layered gradient structures. Vertically, gZCFA exhibits a continuous gradient in fiber diameter from thick to thin, with fibers arranged in layers parallel to the vertical direction. gZCFA exhibits low density (10.3 mg·cm^−3^), high filtration efficiency, and excellent mechanical strength. gZCFA achieves hierarchical filtration, demonstrating superior performance compared to cZCFA and fZCFA (areal density: 22.5 mg·cm^−2^, filtration efficiency: 99.96%, pressure drop: 156 Pa). It exhibits high filtration efficiency for high-temperature gases (>99.95% for PM_0.3_) and a high dust-holding capacity. gZCFA demonstrates outstanding mechanical properties, including a tensile elongation at break of 25.75%, a recoverable compression strain of 80%, and excellent fatigue resistance, withstanding 500 cycles at 50% strain. These exceptional filtration and mechanical properties make gZCFA a promising material for high-temperature flue gas filtration applications.

## Data Availability

The original contributions presented in this study are included in the article/Supplementary Materials. Further inquiries can be directed to the corresponding authors.

## Supplementary Materials

The following supporting information can be downloaded at: https://www.mdpi.com/article/10.3390/nano16060382/s1, Figure S1: Zirconia Precursor Fibers Zirconia Precursor Fibers; Figure S2: gZCFA peeling images along layers; Figure S3: SEM images of the gZCFA cross sections, confirming the existence of a lamellar structure; Figure S4: (a) FE-SEM image and (b) elemental mapping images of the gZCFA fibers; Figure S5: The elemental content is shown in the EDS elemental mapping of gZCFA; Figure S6: (a) XPS (b) XPS spectra of O 1s, Al 2p, Zr 3d, and Si 2p; Figure S7: XRD analyses of gZCFA; Figure S8: The densities, porosities and average pore diameters corresponding to four different diameters of fibers in gZCFA (a) Density. (b) Porosity. (c) average pore size; Figure S9: Thermogravimetric curves of gZCFA precursor and gZCFA in the temperature range 30–1000 °C; Figure S10: Optical image of the filter tester (MFP 3000, PALAS); Figure S11: The filtration performance of sample groups with different fiber diameters; Figure S12: cZCFA (a) Physical image (b) SEM image (c) Fiber diameter distribution; fZCFA (d) Physical image (e) SEM image (f) Fiber diameter distribution; Figure S13: (a) Quality factor of cZCFA with different surface densities under varying gas flow velocities. (b) Quality factor of fZCFA with different surface densities under varying gas flow velocities; Figure S14: The influence of humidity on filtration performance; Figure S15: Optical images of the experimental device we built for high-temperature filtration testing; Figure S16: The particle size distribution of the substances produced by the burning of incense; Figure S17: Filter fatigue test; Table S1: Comparison of the filtration property of the gZCFA with other fiber filtration materials.
