# Supplementary material for: Multi-Scale Gradient Fiber Structure Hierarchical Flexible Ceramic Aerogel for High-Temperature Filtration
Source: Nanomaterials (Basel). 2026 Mar 23;16(6):382. doi: 10.3390/nano16060382 (PMC13029609; doi:10.3390/nano16060382)
Supplement: Supplementary file 1 [file nanomaterials-16-00382-s001.zip › nanomaterials-4198889-supplementary.pdf]

Supplementary information

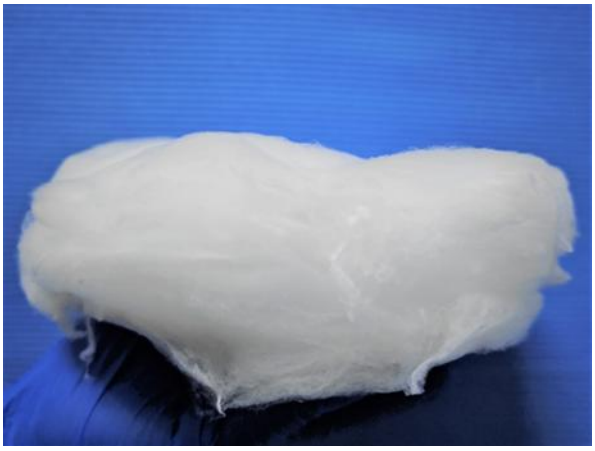

Fig. S1 Zirconia Precursor Fibers Zirconia Precursor Fibers

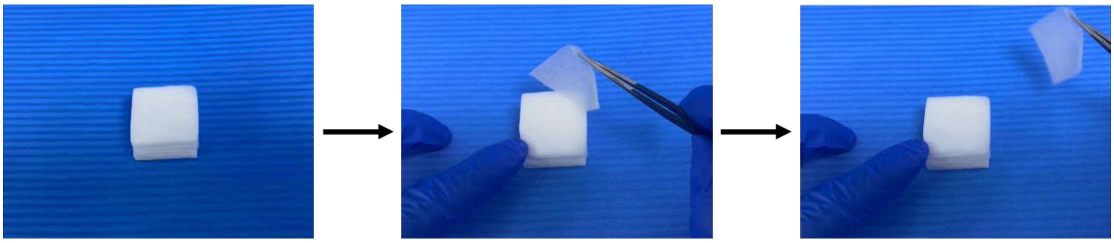

Fig.S2 gZCFA peeling images along layers.

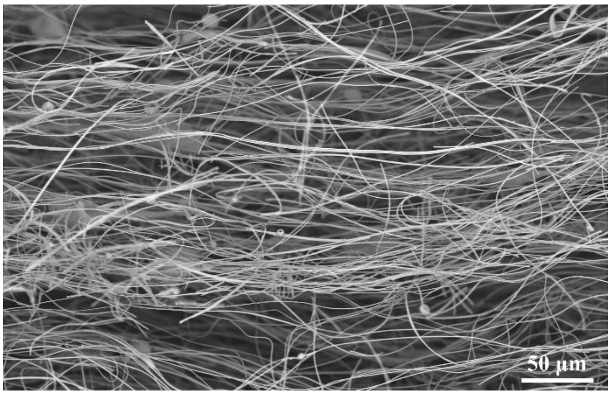

Fig. S3 SEM images of the gZCFA cross sections, confirming the existence of a lamellar structure.

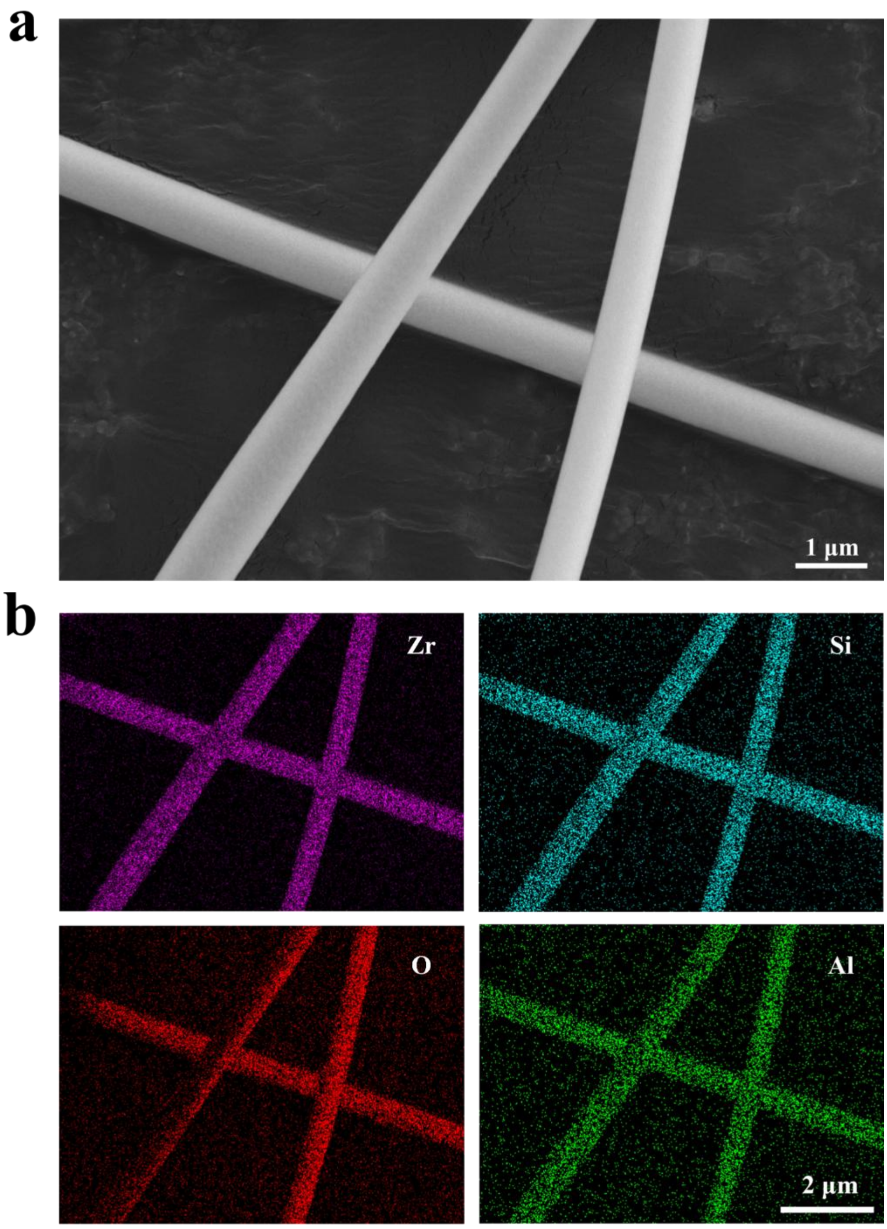

Fig. S4 (a) FE-SEM image and (b) elemental mapping images of the gZCFA fibers.

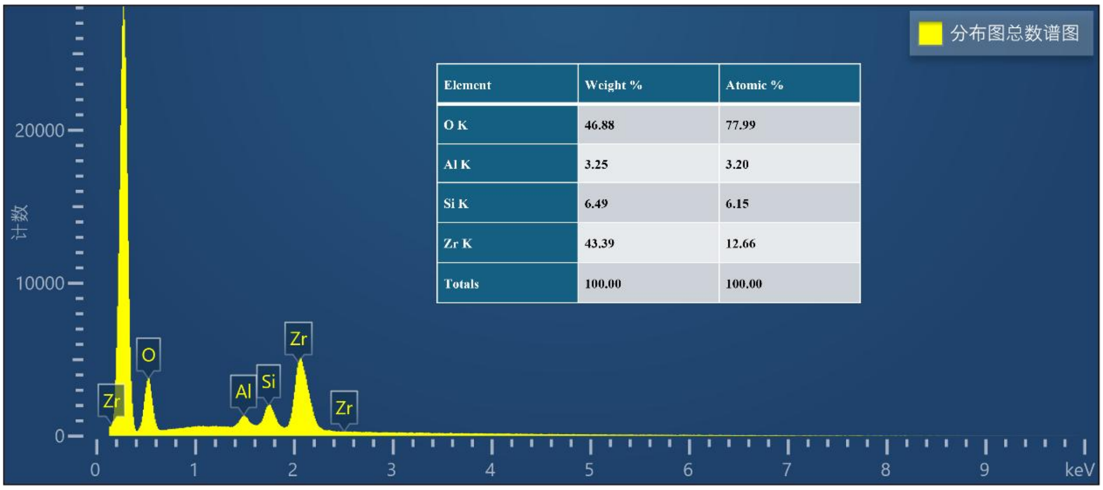

Element	Weight %	Atomic %
O K	46.88	77.99
Al K	3.25	3.20
Si K	6.49	6.15
Zr K	43.39	12.66
Totals	100.00	100.00

Fig. S5 The elemental content is shown in the EDS elemental mapping of gZCFA.

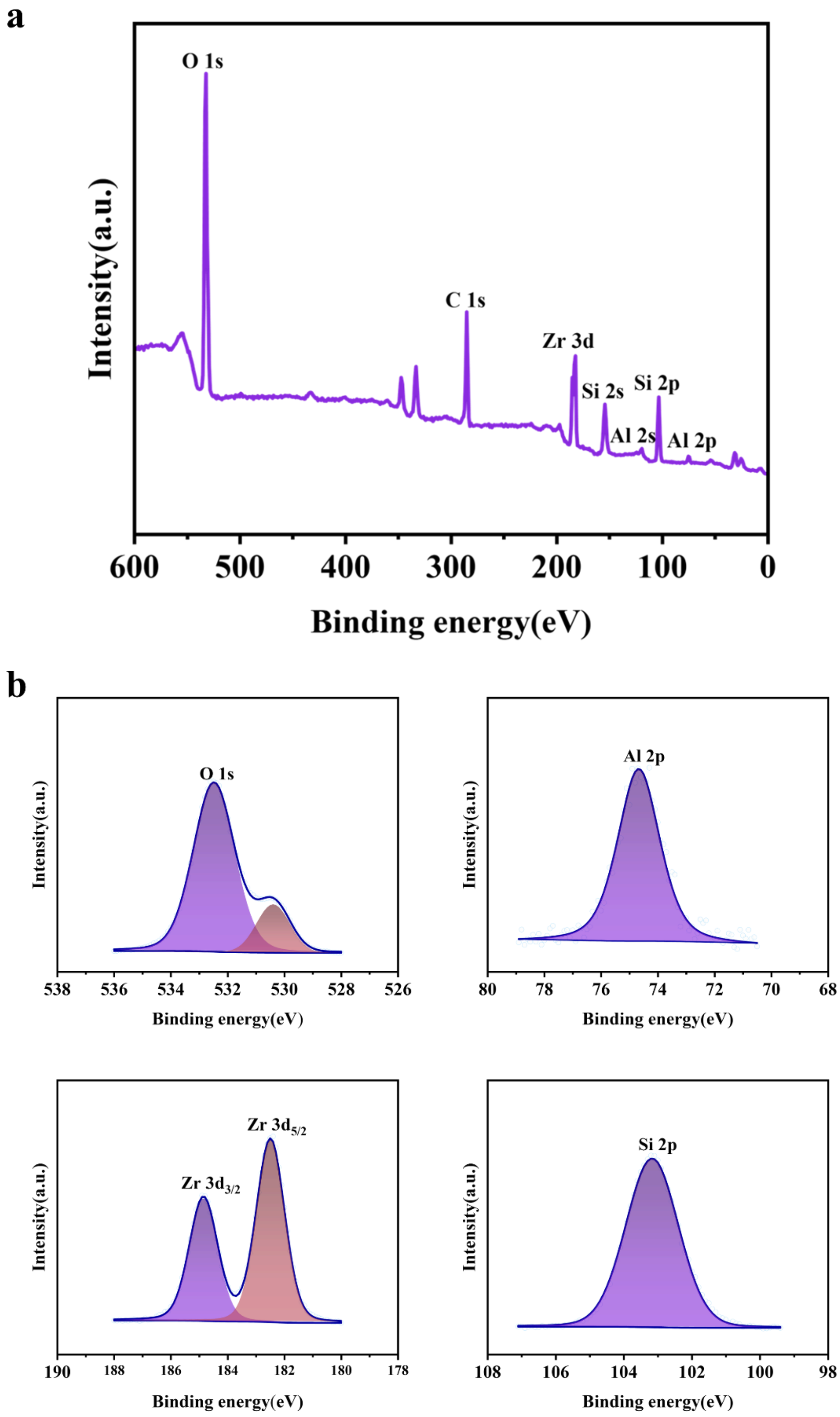

Fig. S6 (a) XPS (b) XPS spectra of O 1s, Al 2p, Zr 3d, and Si 2p.

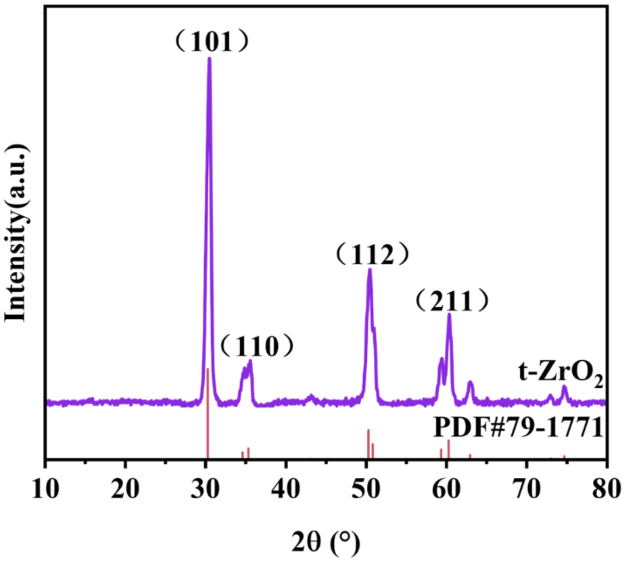

Fig. S7 XRD analyses of gZCFA.

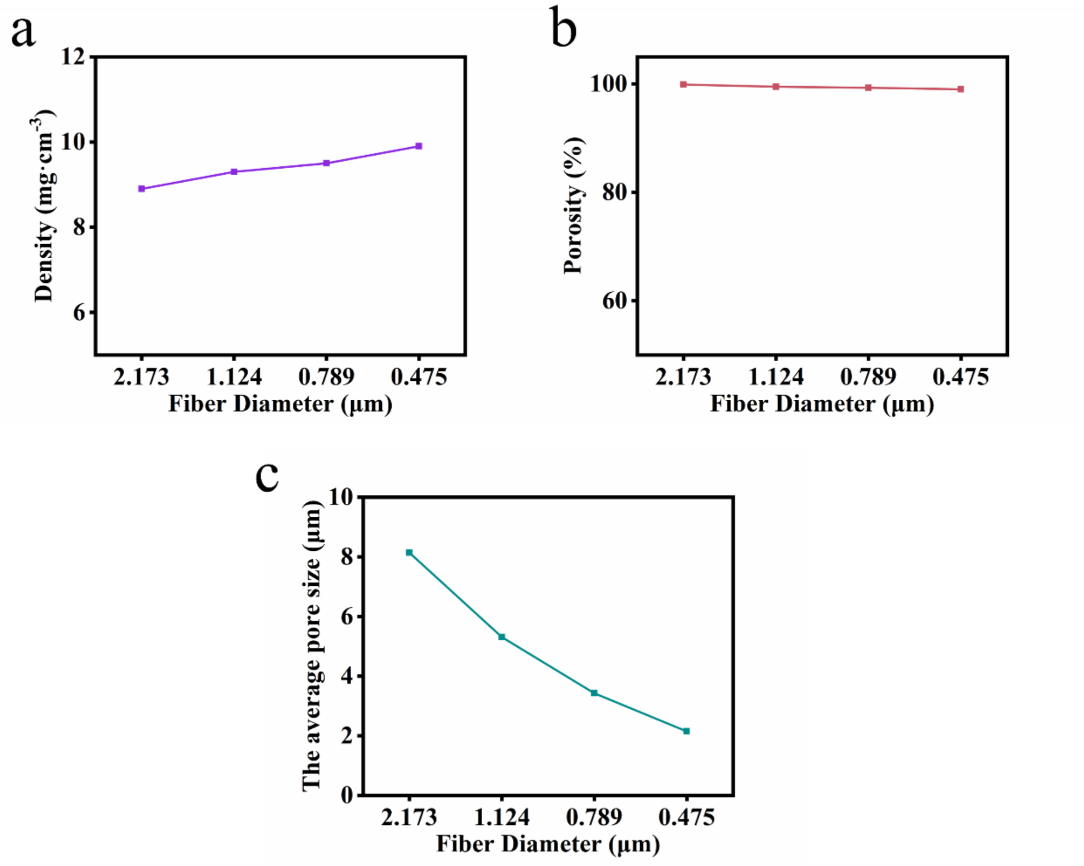

Fig. S8. The densities, porosities and average pore diameters corresponding to four different diameters of fibers in gZCFA (a) Density. (b) Porosity. (c) average pore size.

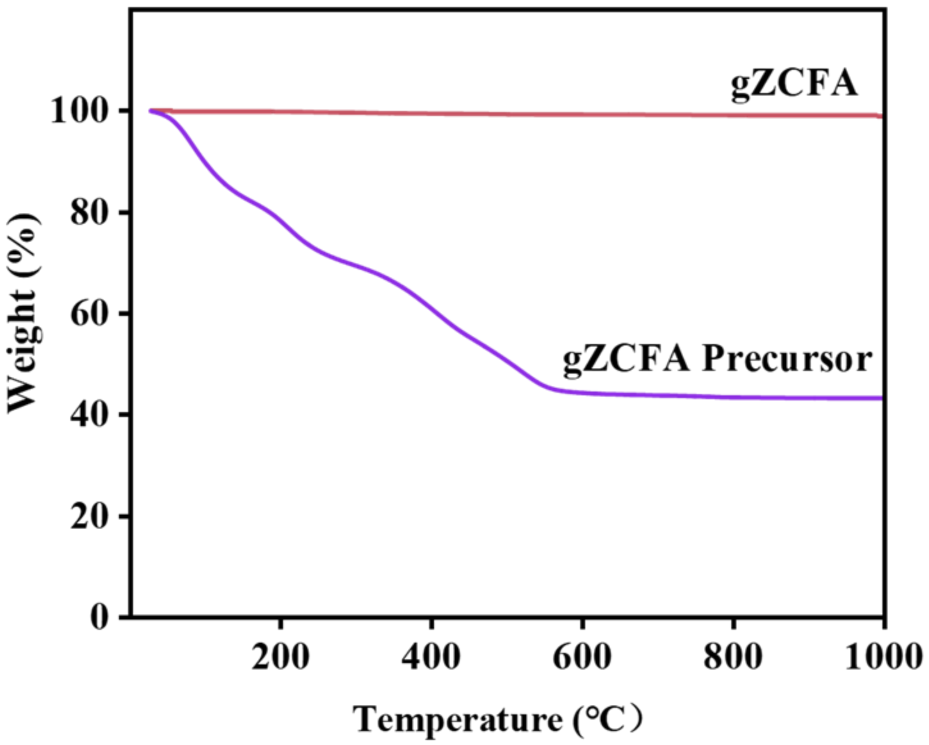

Fig. S9 Thermogravimetric curves of gZCFA precursor and gZCFA in the temperature range 30-1000 °C.

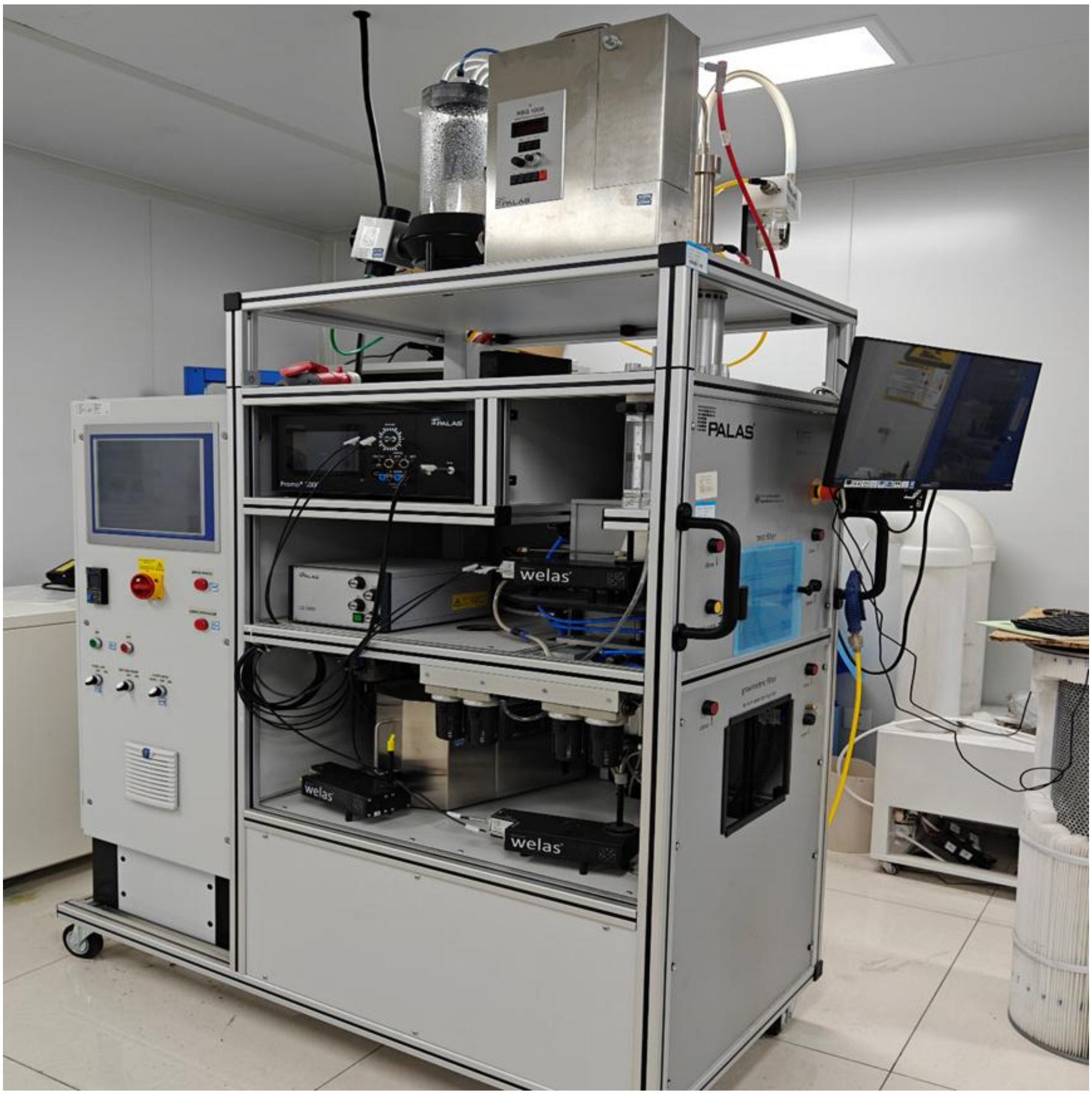

Fig. S10 Optical image of the filter tester (MFP 3000, PALAS).

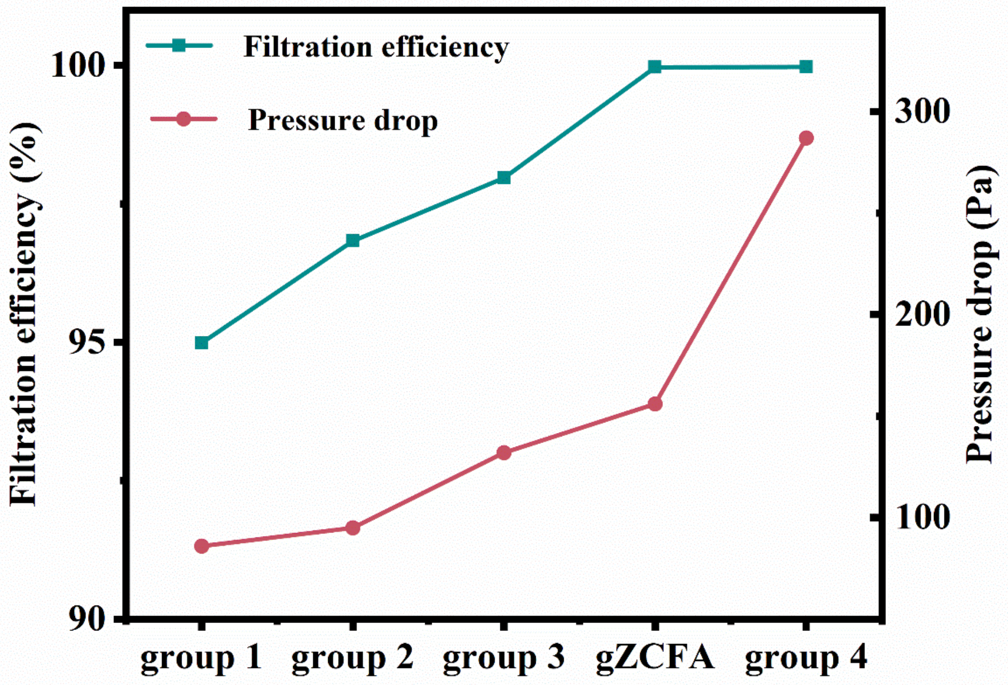

Fig. S11 The filtration performance of sample groups with different fiber diameters.

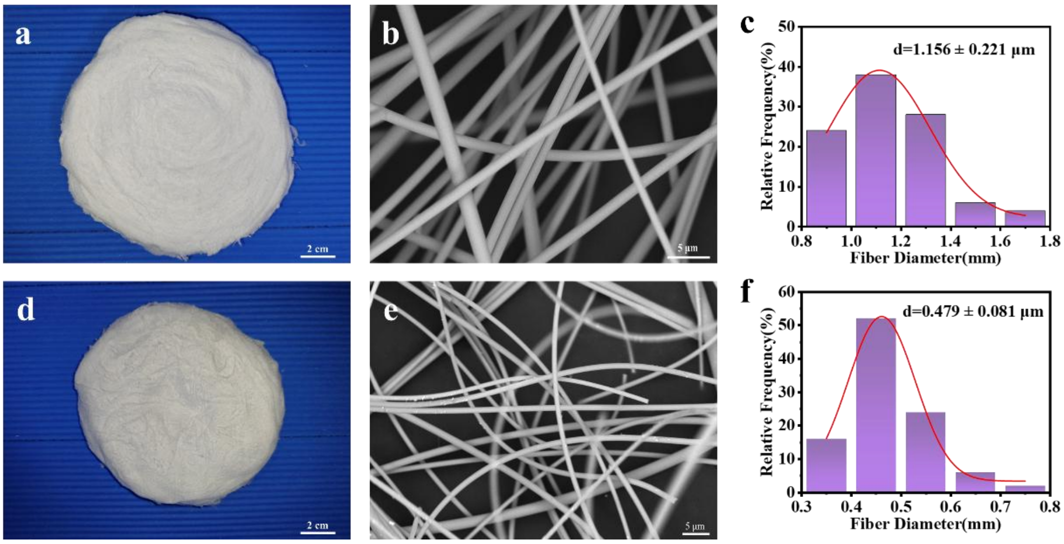

Fig. S12 cZCFA (a) Physical image (b) SEM image (c) Fiber diameter distribution.; fZCFA (d) Physical image (e) SEM image (f) Fiber diameter distribution.

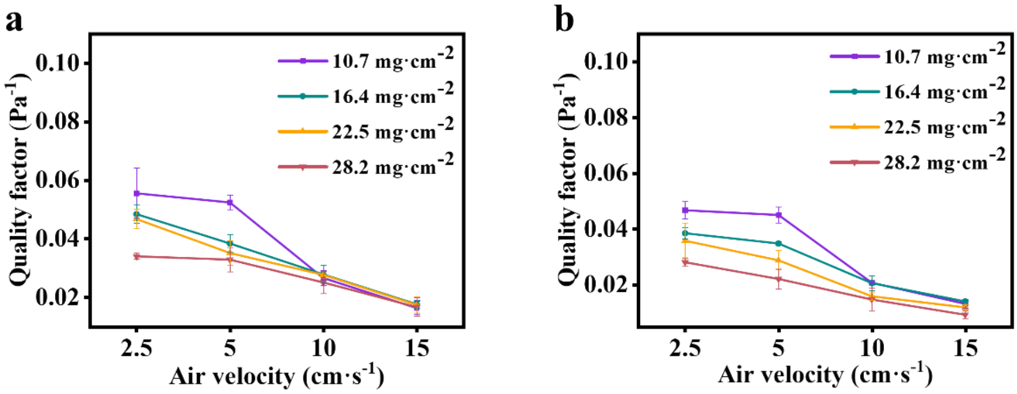

Fig. S13 (a) Quality factor of cZCFA with different surface densities under varying gas flow velocities. (b) Quality factor of fZCFA with different surface densities under varying gas flow velocities.

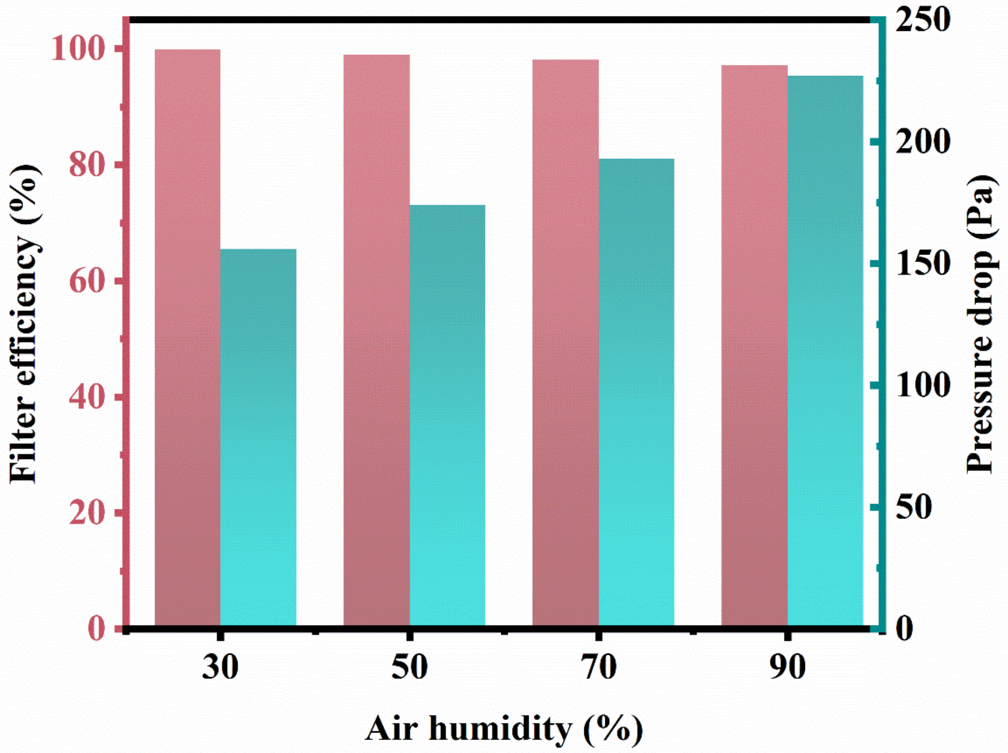

Fig. S14 The influence of humidity on filtration performance.

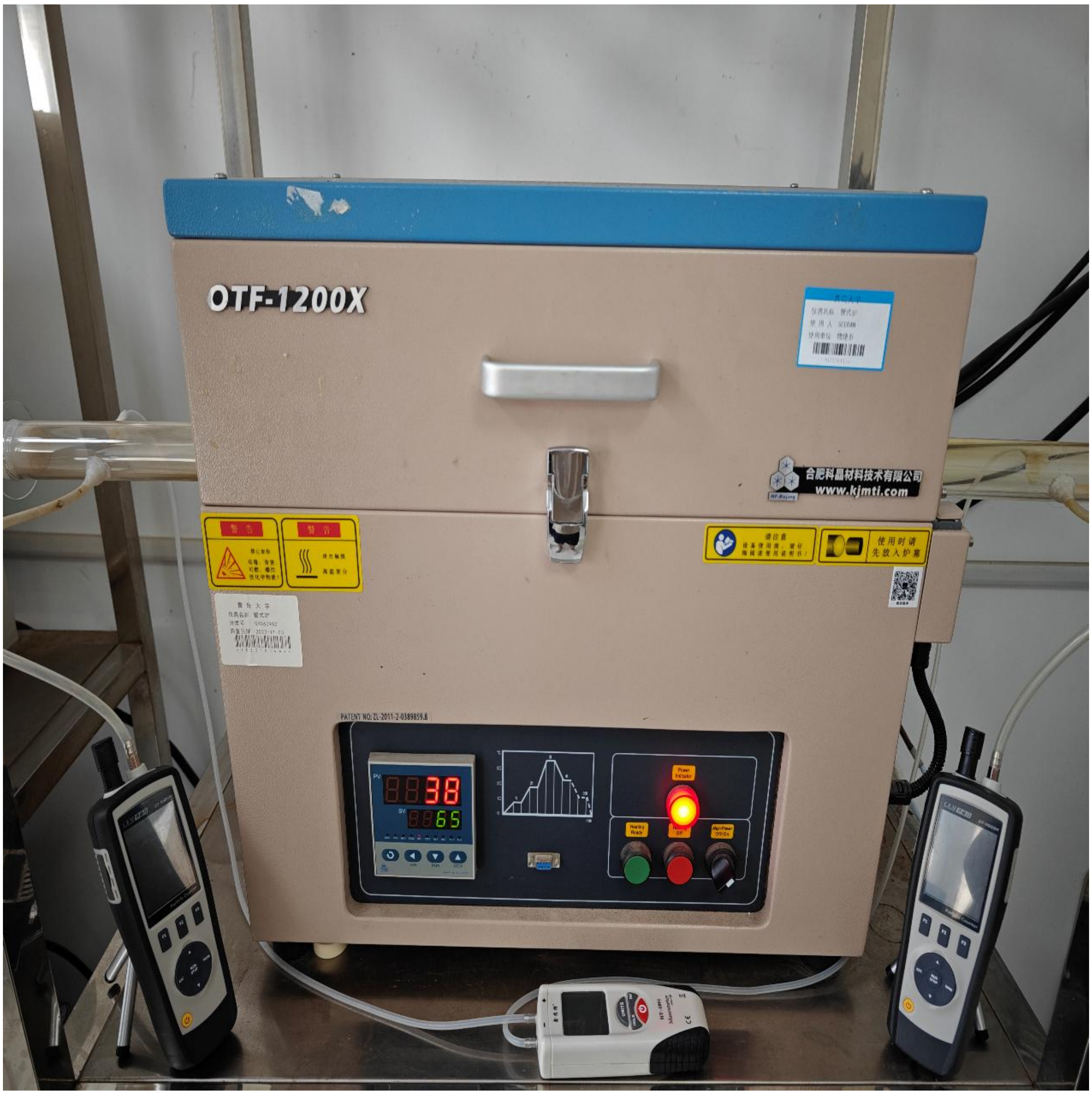

Fig. S15 Optical images of the experimental device we built for high-temperature filtration testing.

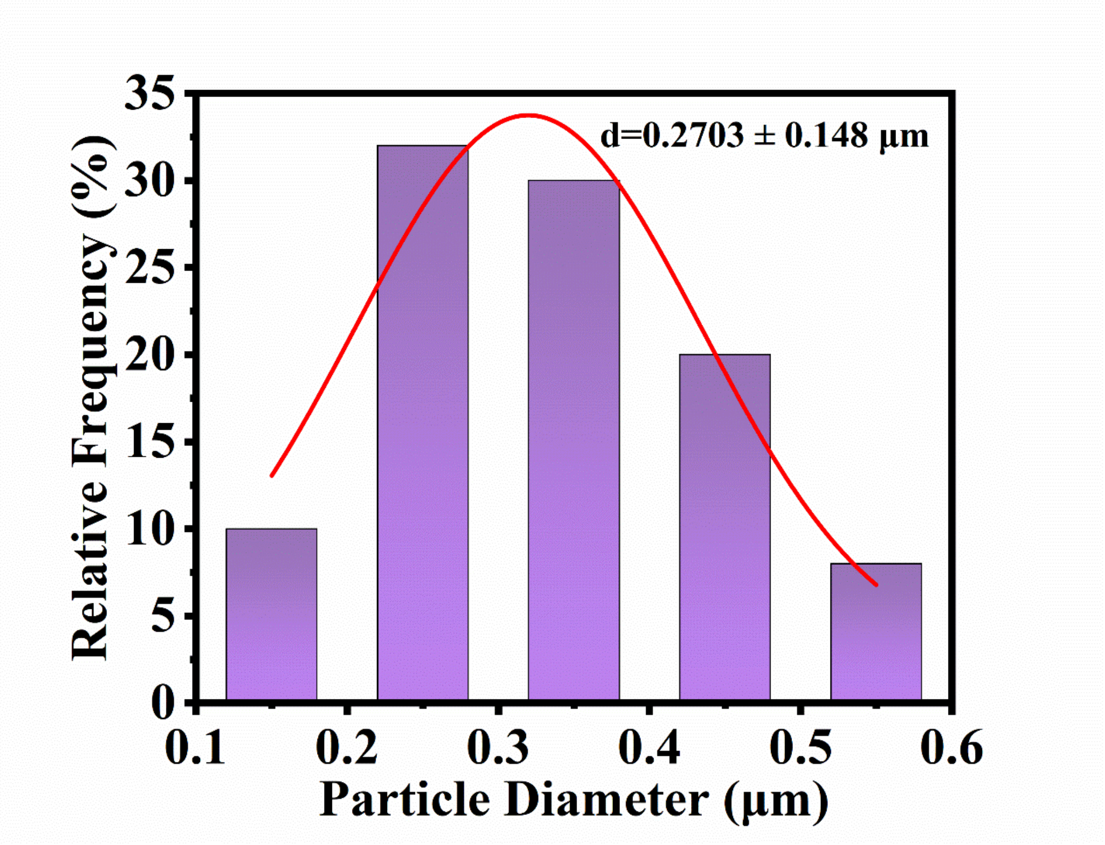

Fig. S16 The particle size distribution of the substances produced by the burning of incense.

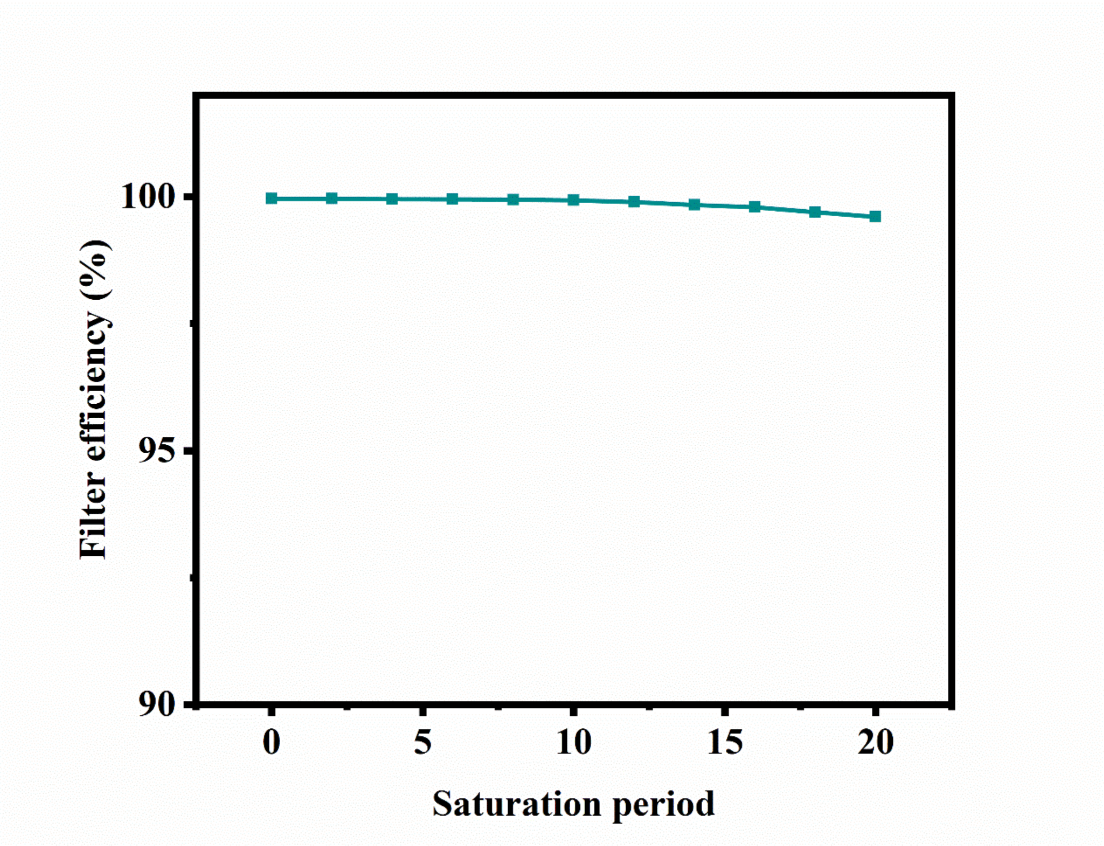

Fig. S17 Filter fatigue test.

Tab. S1 Comparison of the filtration property of the gZCFA with other fiber filtration materials.

**Materials**	**Areal density (mg cm^{-2})**	**Filtration efficiency (%)**	**Pressure drop (Pa)**	**Quality factor (Pa^{-1})**	**Working temperature (°C)**	**References**
gZCFA	22.5	99.96	156	0.051	1100	Our work
$BaTiO_3/Al_2O_3$ ceramic aerogels	22	99.95	166	0.045	800	[1]
YAZ sponge	-	99.4	57	0.09	750	[2]
Mullite fiber sponge	36	99.23	49	0.099	800	[3]
ZrO_2 nanofiber films	20	94.94	136	0.022	1100	[4]
ASZ paper	56	99.56	108	0.050	1100	[5]
PVDF	-	90	18	0.13	-	[6]
PBI	-	98.55	131.2	0.032	400	[7]
PI	-	97.3	83	0.044	400	[8]
PI/ZIF	-	96.6	72	0.047	300	[9]
PVDF/PTFE	-	94.24	18	0.16	-	[10]
PLA-GT	-	96.02	181.43	0.019	180	[11]
N/MFR PI sponge	-	97.13	90	0.0395	400	[12]
3D-MNFG	-	97	92	0,038	100	[13]

References

1. Y. Gao, P.-H. Yu, J. Zhang, G.-D. Zhang, C.-H. Guo, Y.-Q. Zhou, Y.-Z. Long and H. Wu, Compressible Piezoelectric Ceramic Nanofiber Aerogels with Multifunction, Advanced Fiber Materials, 2025, 7 (3):937-949.
2. H. Wang, S. Lin, S. Yang, X. Yang, J. Song, D. Wang, H. Wang, Z. Liu, B. Li, M. Fang, N. Wang and H. Wu, High-Temperature Particulate Matter Filtration with Resilient Yttria-Stabilized ZrO2 Nanofiber Sponge, Small, 2018, 14 (19):1800258.
3. J. Zhu, R. Zhu, Y. Hu and Z. Wang, Low-cost and temperature-resistant mullite fiber sponges with superior thermal insulation and high-temperature PM filtration, Separation and Purification Technology, 2023, 305:122445.
4. Z. Xu, W. Kong, X. Su, Y. Zhai, D. Luo, J. Li, J. Zhao, C. Jia and M. Zhu, Solution blow spun flexible zirconia nanofibers toward high-performance 2D and 3D nanostructures, Ceramics International, 2024, 50 (10):17419-17427.
5. C. Jia, Y. Liu, L. Li, J. Song, H. Wang, Z. Liu, Z. Li, B. Li, M. Fang and H. Wu, A Foldable All-Ceramic Air Filter Paper with High Efficiency and High-Temperature Resistance, Nano Letters, 2020, 20 (7):4993-5000.
6. W. W. F. Leung and Q. Sun, Electrostatic charged nanofiber filter for filtering airborne novel coronavirus (COVID-19) and nano-aerosols, Separation and Purification Technology, 2020, 250:116886.
7. S. Lee, A. R. Cho, D. Park, J. K. Kim, K. S. Han, I.-J. Yoon, M. H. Lee and J. Nah, Reusable Polybenzimidazole Nanofiber Membrane Filter for Highly Breathable PM2.5 Dust Proof Mask, ACS Applied Materials & Interfaces, 2019, 11 (3):2750-2757.
8. Z. Li, J. Song, Y. Long, C. Jia, Z. Liu, L. Li, C. Yang, J. Liu, S. Lin, H. Wang, Y. Liu, M. Fang and H. Wu, Large-scale blow spinning of heat-resistant nanofibrous air filters, Nano Research, 2020, 13 (3):861-867.
9. Z. Hao, J. Wu, C. Wang and J. Liu, Electrospun Polyimide/Metal-Organic Framework Nanofibrous Membrane with Superior Thermal Stability for Efficient PM2.5 Capture, ACS Applied Materials & Interfaces, 2019, 11 (12):11904-11909.
10. S. Wang, X. Zhao, X. Yin, J. Yu and B. Ding, Electret Polyvinylidene Fluoride Nanofibers Hybridized by Polytetrafluoroethylene Nanoparticles for High-Efficiency Air Filtration, ACS Applied Materials & Interfaces, 2016, 8 (36):23985-23994.
11. Y. Sun, S. Eckstein, X. Niu, M. Yermakov, S. Grinshpun, G. Song and G. Sun, Biobased Triesters as Plasticizers for Improved Mechanical and Biodegradable Performance of Polylactic Acid Fibrous Membranes as Facemask Materials, ACS Sustainable Chemistry & Engineering, 2024, 12 (20):7964-7975.
12. T. Zhang, L. Yin, X. Wang, H. Fu, Y. Li, D. Zhang, J. Huang, X. Qian, Y. Lai and S. Zhang, Robust three-dimensional bioinspired honeycomb structured ultra-elastic aerogels for high-temperature cascade filtration applications, Journal of Cleaner Production, 2024, 442:141014.
13. X. Zhao, Y. Bai, G. Fan, H. Guo, P. Shi, W. Cui, X. Jin, Y. Liu, R. Wang and J. He, Large-Scale Fabrication of 3D gradient hierarchical fibrous filter materials with Micro-Submicro-Nanofibers for efficient and Long-Duration air filtration, Separation and Purification Technology, 2025, 362:131867.
